# Telomerase gene expression bioassays indicate metabolic activation of genotoxic lower chlorinated polychlorinated biphenyls

**DOI:** 10.1038/s41598-018-35043-w

**Published:** 2018-11-15

**Authors:** Theresa Vasko, Jenny Hoffmann, Sonja Gostek, Thomas Schettgen, Natalia Quinete, Christian Preisinger, Thomas Kraus, Patrick Ziegler

**Affiliations:** 1 Institute for Occupational, Social and Environmental Medicine, RWTH Aachen University, Aachen, Germany; 2 Southeast Environmental Research Center, Florida International University Florida, Florida, USA; 3 Proteomics Facility, IZKF, RWTH Aachen University, Aachen, Germany

## Abstract

Polychlorinated biphenyls (PCBs) are ubiquitously occurring pollutants with different chemical and toxicological properties. In this study we evaluated blood plasma samples of two PCB-exposed cohorts for their ability to alter telomerase (*hTERT*) gene expression. Blood plasma from PCB-exposed individuals inhibited *hTERT* expression depending solely on the concentration of lower chlorinated PCBs, with the lowest observed adverse effect level (LOAEL) at a plasma concentration between 0.5 and 2 µg/L of LC PCBs. Individual OH-metabolites derived from the WHO indicator congeners PCB 28 and PCB 101 mimicked these effects on *hTERT* expression *in vitro* with high toxicity, including DNA damage. However, by the combination of different OH-metabolites, the bio effective PCB concentration was reduced and the respective effects on *hTERT* expression could be increased. At a concentration which showed no toxic activity in MTT assay, *hTERT* inhibition reflected the interference of OH-PCBs with the mitochondrial respiratory chain, which could lead to the production of reactive oxygen species (ROS). As individual OH-metabolites already showed a much stronger inhibition of *hTERT* gene expression at a lower concentration than their parental compounds, the *hTERT* gene expression bioassay described in this study seems to indicate metabolic activation of LC PCBs rather than the mere effect of LC PCBs on their own. In summary, this study provides dose-response linkages between effects of lower chlorinated PCBs and their concentrations in human plasma.

## Introduction

Polychlorinated biphenyls (PCBs) are abundantly present organochlorine pollutants eliciting adverse biological effects. PCBs are categorized according to their degree of chlorination (low and high chlorinated PCBs), substitution pattern (chlorine substituent in ortho-, meta-, para-position), or by producing similar effects as other chemicals within biological systems (dioxin-like PCBs). Non-orthochlorinated PCBs have a coplanar spatial structure and contribute substantially to the toxicity of PCB mixtures^1^. The International Agency for Research on Cancer (IARC) classified PCBs as carcinogenic to humans (Group 1)^2^. IARC further stresses the importance of metabolic activation for the carcinogenic effects of PCBs^3^. Different isoforms of hepatic CYP450 monooxygenases sequentially convert PCBs into mono- and dihydroxy metabolites (OH-PCBs). The underlying enzymatic transformations are partially subject to high stereo- and region-specificity, which can result in a multitude of different metabolites^4,5^. Low degree of chlorination and non-chlorinated ortho-positions in coplanar PCBs accelerate their enzymatic conversion^6^. Although OH-PCBs are expected to be combined with glucuronic acid, glutathione or sulphate and excreted^7,8^, some of them are strongly retained in human blood by enterohepatic circulation^9^. The metabolism of PCB congeners therefore not only results in the formation of a very large number of different OH-PCBs, but also of OH-PCB congeners with partially higher toxicity and/or higher half-lives than their parental compounds^10–12^. In peripheral tissue, OH-metabolites of LC PCBs can produce catechols or hydroquinones^13^. Through autoxidation (autocatalytic oxidation in the presence of oxygen), peroxidases (POX) or the prostaglandin-H synthase (PHS), catechols and hydroquinones can be further modified to ortho- or para-quinones. These reactive arene oxides can covalently bind to biological macromolecules and form protein-, RNA- and DNA-adducts^14^. The electron transfer associated with the oxidation of OH-PCBs can lead to the formation of free oxygen radicals. Genotoxicity, mediated by adduct formation and generation of reactive oxygen species (ROS) is regarded as an important mechanism for the carcinogenic effect of LC PCBs^15^.

Several studies have shown that PCBs can influence telomerase (*hTERT*) and telomere length (TL) *in vivo* and *in vitro*. Leukocyte telomere length were prolonged in individuals after long-term exposure to low doses of non orthochlorinated PCBs and DL PCBs^16–18^. In contrast to these observations, *in vitro* studies with individual airborne PCB congeners showed a reduced TL and *hTERT* enzyme activity in liver and keratinocyte cell lines^13,19^. In our previous experiments^20^, we found a highly significant reduction of age-adjusted TL in peripheral blood lymphocytes of high-level PCB exposed individuals (∑ indicator PCBs up to 236 µg/L plasma^21^) where a dependency of telomere shortening on the exposure level of LC PCBs could be shown. Telomere shortening in lymphocytes can lead to replicative senescence, a terminal state which correlates with the progressive loss of immunological memory, a phenomenon known as immunosenescence. Accelerated onset of immunosenescence as seen for instance in chronic viral infections, compromises T-cell mediated immunity^22^. Immunosenescence has been related to higher infection rates, cancer and age-related diseases, and these events have also been associated with the exposure to PCBs in the past^2,23,24^. Accelerated telomere shortening in lymphocytes could thus contribute to the immunosuppressive activity of PCBs and explain some of the longterm effects of PCB exposure on the adaptive immune system.

Here we address the question to what extent the inhibition of *hTERT*, the telomere extending enzyme, can provide specific information about the extent of PCB contamination and whether it can be used to draw conclusions on PCB metabolic activation and PCB induced adverse health effects. We therefore made use of blood plasma samples of two characterized cohorts of PCB exposed individuals and focused on the WHO indicator congeners, 2,4,4′-trichlorobiphenyl (PCB 28) and 2,2′,4,5,5′-pentachlorobiphenyl (PCB 101). Furthermore we extended findings from blood plasma samples in a model cell culture system using hydroxylated metabolites of PCB 28 and PCB 101.

## Results

### Inhibition of *hTERT* gene expression by blood plasma of PCB exposed individuals

**Figure 1:**
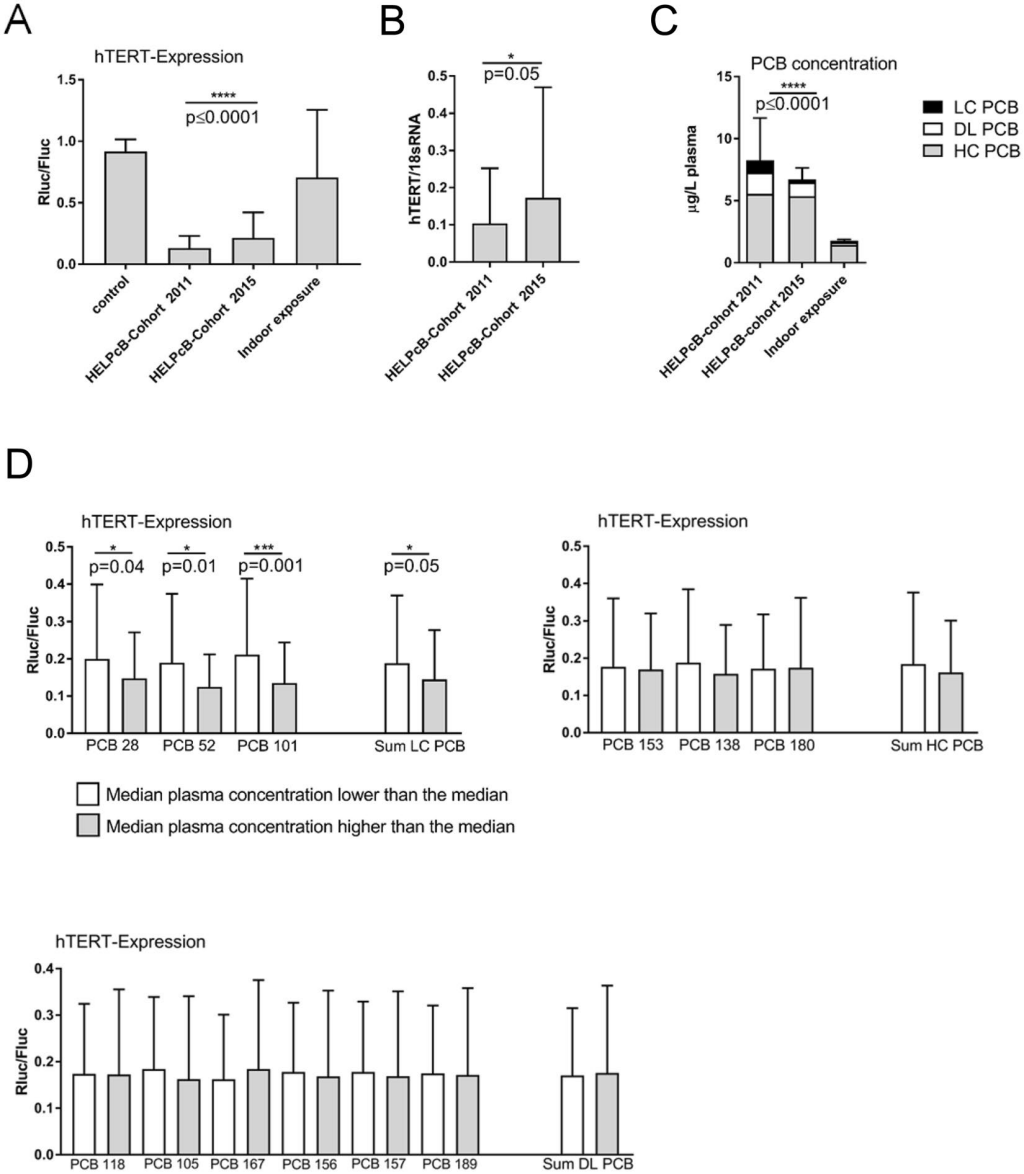
Inhibition of *hTERT* gene expression by blood plasma of PCB exposed individuals. (**A**) Tert^+^ B6B5.1 cells were incubated with longitudinally collected plasma samples from the HELPcB-cohort (N = 92 per time point; each with three technical replicates) or with plasma samples from individuals exposed to PCB via indoor air (N = 105; each with three technical replicates). After 48 hours of incubation, luciferase activities were determined. Statistical analysis was performed by a Wilcoxon matched-pairs signed rank test. Statistically significant differences are indicated (*P = 0.05; ****P < 0.0001). (**B**) PBMCs from healthy donors (0 RhD negative) were stimulated with tetanus toxoid for five days, reseeded and incubated in the presence of antigen- and PCB-containing plasma samples from the HELPcB-cohort as described in A. After 48 hours of incubation, *hTERT* gene expression was assessed by qRT-PCR. Statistical analysis was performed by a Wilcoxon matched-pairs signed rank test. (**C**) Mean plasma levels for Σ higher chlorinated PCBs (grey), Σ dioxin-like PCBs (white) and Σ lower chlorinated PCBs (black) from longitudinally collected samples (HELPcB-cohort) and from individuals exposed to PCB via indoor air. Statistical analysis was performed by a Wilcoxon matched-pairs signed rank test. SD and statistically significant differences are given for Σ lower chlorinated PCBs (****P < 0.0001). (**D**) Mean ± SD ratio of *hTERT* expression (N = 184) for individual indicator PCBs depending on plasma concentration. HC PCBs, DL PCBs and LC PCBs were separated using the median value as cut off. Statistical analysis was performed by a Mann-Whitney test. Statistically significant differences are indicated.

We have previously shown, that blood plasma samples containing high concentrations of PCB 28 inhibit the expression of telomerase (*hTERT*), the telomere-extending enzyme^20^. In order to further evaluate these findings in depth we made use of tert^+^ B6B5.1 cells, targeted with a transgenic bacterial artificial chromosome (BAC) reporter containing a *Renilla* (*Rluc*) and a *Firefly* (*Fluc*) luciferase expression cassette under the control of the *hTERT* and *CRR9* promoter^25^. The resulting ratio of *Rluc* to *Fluc* activity has been shown to be a reliable assessment of *hTERT* promoter activity^25^. When incubated with longitudinally collected blood plasma samples from individuals with a high PCB body burden due to occupational exposure (HELPcB-cohort)^21^, the capability of plasma to inhibit *hTERT* gene expression in tert^+^ B6B5.1 cells was reduced over time, whereas plasma samples collected in 2011 inhibited *hTERT* expression by a factor of 7 (mean *Rluc/Fluc*-ratio = 0.13) as compared to untreated controls (mean *Rluc/Fluc*-ratio = 0.92), the suppressive effect of plasma was less pronounced in 2015 (mean *Rluc/Fluc*-ratio = 0.21; 4 fold inhibition as compared to controls) (Fig. 1A). Similar results were obtained when tetanus toxoid stimulated peripheral blood mononuclear cells (PBMCs) were incubated with blood plasma from the HELPcB-cohort and *hTERT* mRNA levels were assessed by quantitative RT-PCR (qRT-PCR). Here plasma samples collected in 2011 caused a significantly stronger inhibition of *hTERT* expression levels (mean *hTERT*/*18S rRNA*-ratio = 0.1) than the corresponding plasma samples collected in 2015 (mean *hTERT*/*18S rRNA*-ratio = 0.16) (Fig. 1B). In contrast, blood plasma collected from a cohort of residents exposed to PCBs via indoor air scarcely influenced *hTERT* gene expression (mean *Rluc/Fluc*-ratio = 0.71) as compared to controls (Fig. 1A). Corresponding mean PCB levels in the plasma of the individuals were 1.44 μg/L for the sum of higher chlorinated (HC) PCBs, 0.218 μg/L for the sum of dioxin-like (DL) PCBs and 0.109 μg/L for the sum of lower chlorinated (LC) PCBs (Fig. 1C). With the exception of the sum of LC PCBs, residents therefore did not show significantly elevated blood PCB levels compared to the general population. In contrast, high internal exposures for PCBs have been described for individuals of the HELPcB-cohort^26^. Plasma PCB levels of longitudinally collected samples of this cohort showed no significant difference in the level of HC PCBs at two different time points (mean concentration HC PCB 2011 = 5.55 μg/L vs mean concentration HC PCB 2015 = 5.36 μg/L, corresponding to 3,4% decrease, p = 0.63,), whereas levels of DL PCBs (mean concentration DL PCB 2011 = 1.75 μg/L vs mean concentration DL PCB 2015 = 1.11 μg/L, corresponding to 36,6% decrease; p = 0.13) and LC PCBs (mean concentration LC PCBs 2011 = 0.95 μg/L vs mean concentration LC PCBs 2015 = 0.25 μg/L, corresponding to 73,7% decrease, p = 0.015) decreased over time (Fig. 1C). We therefore conclude that high PCB plasma levels inhibit *hTERT* gene expression in transformed tert^+^ B6B5.1 cells as well as in antigen-driven lymphoproliferation (Fig. 1B). The inhibition of *hTERT* expression within plasma samples from the HELPcB-cohort decreases over time, paralleling the decrease in levels of DL and LC PCBs.

To further analyze the suppressive effects of blood plasma from the HELPcB-cohort, we pooled data from longitudinally collected samples and analyzed *hTERT* expression in plasma dependent on concentrations of LC, HC and DL PCBs. The extent of *hTERT* inhibition in tert^+^ B6B5.1 cells exposed to plasma was dependent on both the PCB congener profile as well as the concentration of PCBs found in plasma of individuals (Fig. 1D). Whereas the plasma concentration of HC PCBs and DL PCBs had no effect on *hTERT* expression, a high plasma concentration of LC PCBs led to a more pronounced and significant inhibition of *hTERT* expression as compared to a low concentration of LC PCBs (Fig. 1D). We therefore conclude that the capability of PCB contaminated plasma from the HELPcB-cohort to inhibit *hTERT* expression depends on the concentration of LC PCBs and decreases with the elimination of LC PCBs due to biotransformation.

### Plasma level-response relationship of LC PCBs in tert^+^ B6B5.1 cells

**Figure 2:**
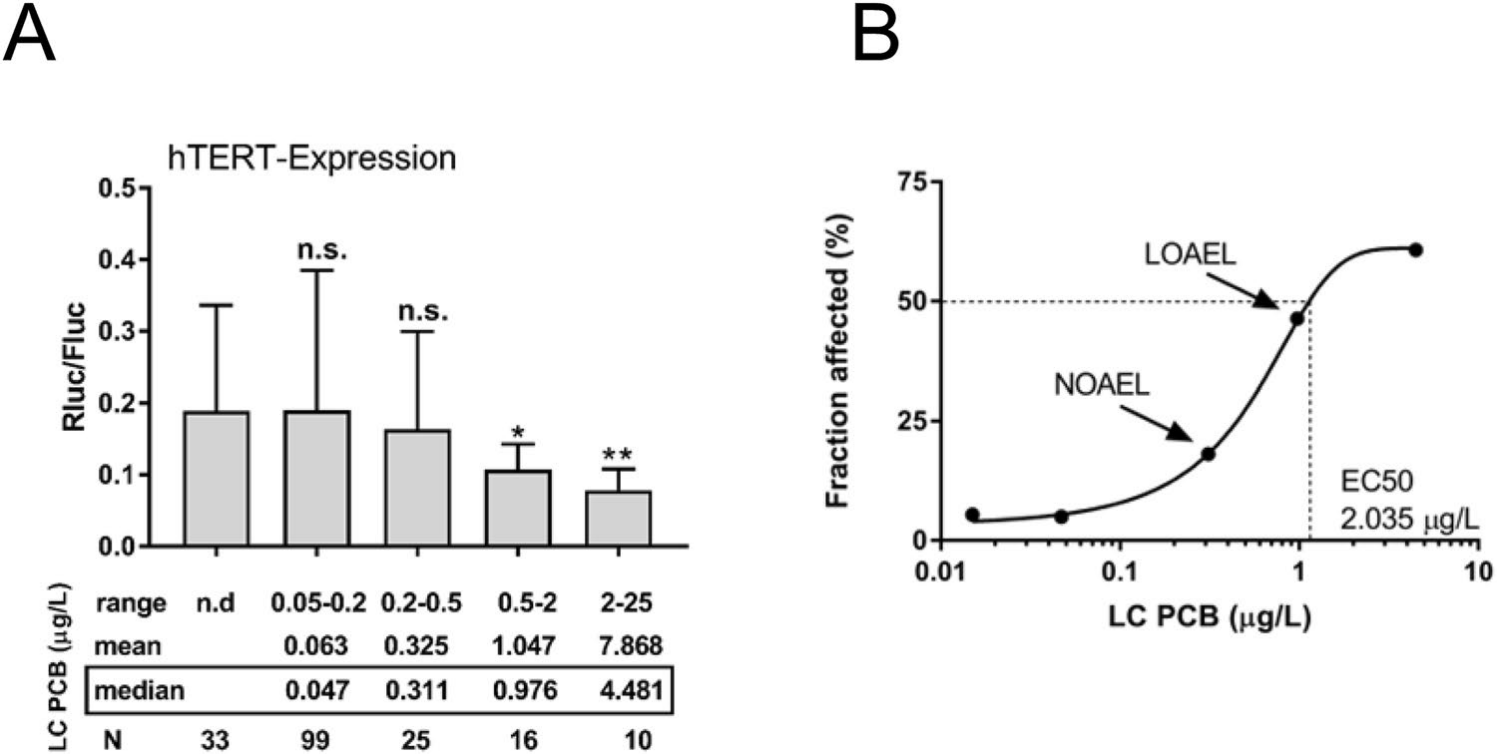
Plasma level-response relationship of LC PCBs in tert + B6B5.1 cells. (**A**) Individual samples of the HELPcB-cohort were divided into five groups depending on plasma levels of LC PCBs. Mean *Rluc/Fluc*-ratio within each group was compared to the group with no detectable levels of LC PCBs. Statistical analysis was performed by a Mann-Whitney test. Statistically significant differences are indicated (*P = 0.05; **P = 0.003). **(B**) The plasma level-response logarithmic curve of LC PCBs in tert^+^ B6B5.1 cells.

As of today, dose-response linkages between adverse health effects of LC PCBs in humans and their concentrations in the human body are not available. In order to model the effects of LC PCBs for *hTERT* gene expression, we divided individuals of the HELPcB-cohort into five groups according to plasma concentrations of LC PCBs (Fig. 2A). Mean *Rluc/Fluc*-ratio of each group was statistically compared to the group of individuals without detectable levels of LC PCBs (mean *Rluc/Fluc*-ratio = 0.19) (Fig. 2A). *hTERT* inhibition was significant at a minimum plasma concentration range between 0.5 and 2 μg/L LC PCBs (mean *Rluc/Fluc*-ratio = 0.1), representing the lowest observed adverse effect level (LOAEL) within the HELPcB-cohort (Fig. 2A). In a dose-response curve we related the median plasma concentration of LC PCBs in each group to the affected fraction of B6B5.1 cells susceptible to *hTERT* inhibition. The resulting plasma concentration-response relationship for the five concentrations yielded a typical sigmoidal curve, with the entire range of response data considered (Fig. 2B). These results imply, that *hTERT* gene expression in tert^+^ B6B5.1 cells allows for discriminating between high and low exposure levels of LC PCBs in human plasma samples.

### *hTERT* inhibition and toxicity by activation of LC PCBs

**Figure 3:**
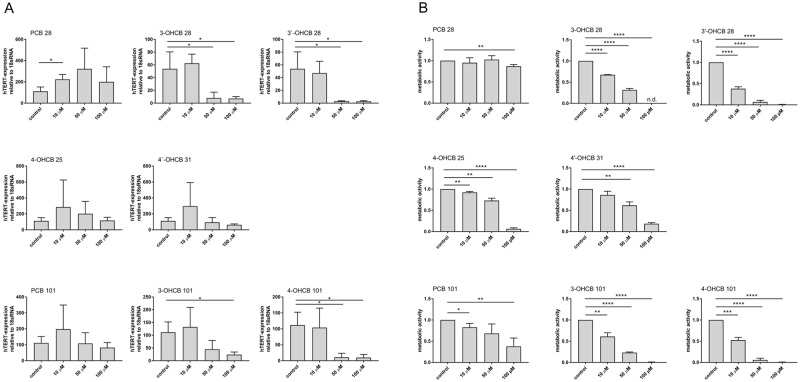
*hTERT* inhibition and toxicity by activation of LC PCBs. (**A**) Jurkat T cells were treated with increasing concentrations of parent PCBs or OH-metabolites for 48 hrs. as indicated. *hTERT* mRNA expression was evaluated by RT-PCR with expression levels normalized against 18S rRNA. Mean ± SD of three different experiments each with a total of three replicates are shown. Statistical analysis was performed by an unpaired, two-tailed Student’s t-test. Statistically significant differences are indicated (*P < 0.05). (**B**) Jurkat T cells were incubated as described in (**A**). Mitochondrial and cellular viability were assessed by means of MTT-assay performed as described. Mean ± SD of three different experiments each with a total of three replicates are shown. Statistical analysis was performed by an unpaired, two-tailed Student’s t-test. Statistically significant differences are indicated (*P < 0.05; **P < 0.01; ***P < 0.0005; ****P < 0.0001).

A variety of PCB toxication processes involve or depend on the metabolism of parent PCBs or their metabolic progeny^6^. We have previously shown that PCB 28 forms four and PCB 101 forms two hydroxylated PCB metabolites in the plasma of PCB exposed individuals^27^. With respect to the inhibition of telomerase gene expression, this could mean that the cumulative effect of hydroxy-metabolites emanating from PCB 28 and PCB 101 contribute to the plasma level-response relationship of LC PCBs in the HELPcB-cohort. Therefore, each OH-PCB (structural formulas in Supplementary Fig. 1) as well as the parent compound was tested at three different concentrations to inhibit *hTERT* expression in human Jurkat T cells (Fig. 3A). Changes in *hTERT* expression levels between the control and treated Jurkat T cells were normalized to 18S mRNA levels and then calculated by the 2-ΔΔct method. In parent PCB 28 treated cultures, *hTERT* expression increased above control levels at all concentrations tested (10–100 µM), whereas the PCB 28 derived metabolites 3-OHCB 28 and 3′-OHCB 28 decreased *hTERT* expression reaching almost 100% inhibition at 50 µM. The other two congeners of PCB28, 4-OHCB 25 and 4′-OHCB 31 stimulated *hTERT* expression at 10 μM or 10 µM and 50 µM (4-OH-CB25) with only 4′-OHCB 31 inhibiting *hTERT* expression below control levels at 50 µM and 100 µM concentration. Similarly, the parent PCB 101 stimulated *hTERT* expression at 10 µM, was equal to control levels at 50 µM and reached a level below 75% of control at 100 µM concentration. Both metabolites of PCB 101, 3-OHCB 101 and 4-OHCB 101, inhibited *hTERT* expression almost completely at 50 µM and 100 µM concentrations, with 4-OHCB 101 starting at 10 µM and showing overall more significant effects. In summary, the OH-derivatives 3-OHCB 28 and 3′-OHCB 28, as well as 3-OHCB 101 and 4-OHCB 101 are more potent in inhibiting *hTERT* gene expression than their parent PCBs and the PCB 28 derivatives 4-OHCB 25 and 4′-OHCB 31. To see if OH-PCBs also display cytotoxic activity towards Jurkat T cells, we assessed the influence of PCB 28 and PCB 101 derived OH-PCBs on metabolic activity using MTT assays (Fig. 3B). When cells were treated with OH-derivatives 3-OHCB 28 and 3′-OHCB 28, as well as 3-OHCB 101 and 4-OHCB 101, a concentration of 100 µM reduced metabolic activity below 10% of control values. At a concentration of 50 µM, the inhibition of metabolic activity was variable between congeners and reached a level below 50% of control values. At a concentration of 10 µM, the influence of all congeners on metabolic activity was less pronounced. These results therefore suggest, that depending on concentration, the effects of hydroxylated PCBs on *hTERT* gene expression and metabolic activity were higher than those of parent PCBs. Assuming that in MTT assays the conversion of tetrazole to formazan depends on the number of viable cells, these results further imply, that the effects of individual OH-PCBs on *hTERT* gene expression in Jurkat T cells mostly reflect a lower number of living cells. However, it must be emphasized here that the concentrations used for these *in vitro* experiments are several log levels higher than the concentrations measured in the blood plasma of the HELPcB-cohort. This should be taken into consideration when drawing conclusions on the acute toxicity of the individual metabolites.

### DNA damage induced by activation of LC PCBs

**Figure 4:**
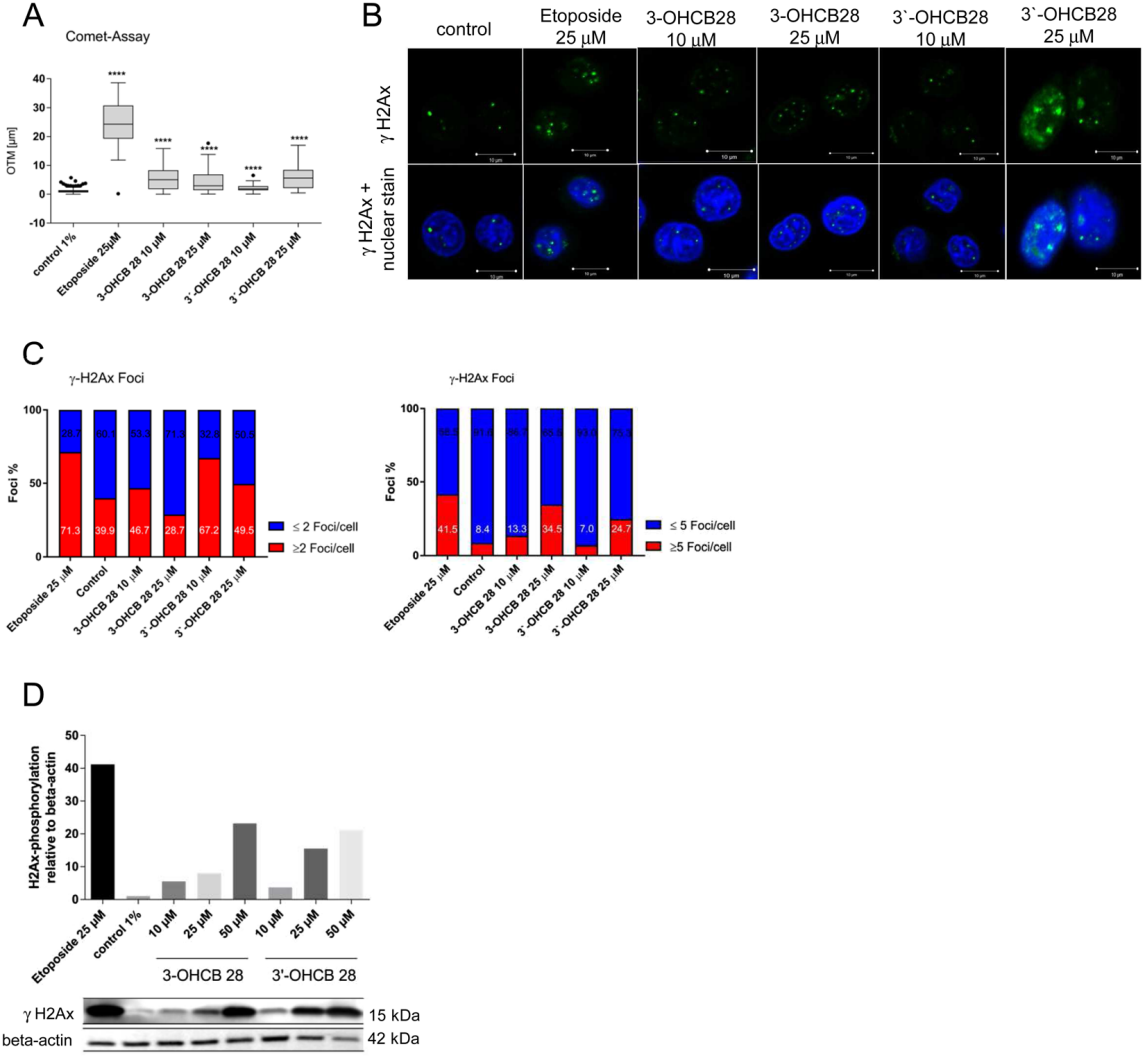
DNA damage induced by activation of LC PCBs. (**A**) Jurkat T cells were incubated with increasing concentrations of 3-OHCB 28 and 3′-OHCB 28 and etoposide as positive control. After 48 hrs. of incubation a comet-assay was performed. Olive tail moments are represented in box plots. Differences of the medians in comparison to negative control were investigated using Kruskal-Wallis, followed by a Dunn’s post-hoc test for multiple comparisons (****P < 0.0001). (**B**) Representative immunofluorescence images of Jurkat T cells treated as described in A. H2Ax-phosphorylation (green, Alexa Fluor 488) was merged with the nuclear counterstain DAPI (blue). (**C**) Quantitative evaluation of confocal images from Jurkat T cells. At least 58 cells per sample were counted. Left, percentage of cells with <2 or >2 foci/cell. Right, percentage of cells with <5 or >5 foci/cell. (**D**) Western blot analysis of γH2Ax in Jurkat T cells treated with 3-OHCB 28 and 3′-OHCB 28 and etoposide for 48 hours. Fold differences of H2Ax-phosphorylation were determined by densitometric analysis.

Our results on toxicity indicate that the tested OH-PCBs display a higher cytotoxicity than their parental compounds at high concentrations. Based on these findings and reports from others^15^, we asked if OH-PCB induced cytotoxicity is caused by DNA damage that occurred in treated cells. The generation of reactive PCB metabolites, ROS or PCB dysregulated proteins can have a major impact on the integrity of DNA. We therefore assessed the ability of 3-OHCB 28 and 3′-OHCB 28, the major metabolites in the HELPcB-cohort, to induce DNA double strand breaks (DSB). We exposed Jurkat T cells for 48 hours to 3-OHCB 28 and 3′-OHCB 28, after which DNA damage was determined with a neutral COMET assay, which measured double strand breaks (DSB) in individual cells. The olive tail moment was calculated and used to quantify DNA damage. As shown in Fig. 4A, both 3-OHCB 28 and 3′-OHCB 28 induced DSB, with DNA damage from 3′-OHCB 28 increasing dose-dependently. DNA fragmentation correlated especially for 3′-OHCB 28 with the duration of action and increase in concentration.

A very early, indirect response to DSB in the cell is the phosphorylation of the histone H2Ax at serine 139 (γH2Ax), which happens immediately after the occurrence of DNA damage and can be detected as foci in the cell nucleus by using a phosphospecific antibody. The confocal microscopy images of Jurkat cells (Fig. 4B) show increased foci in the nuclei upon 3-OHCB 28 and 3′-OHCB 28 incubation. The images show that higher substance concentration correlated with increased focus formation. Since basal DNA damage also occurs in untreated cells, a distinction was made between those with more than two foci per nucleus and those with less than two foci per nucleus. The foci of at least 58 cells were counted for quantification. After differentiation of the cells into those containing more and less than two or five foci, the individual ratios were calculated in each approach. We determined more than five foci per cell to be severe DNA damage. The proportion of these cells is shown in Fig. 4C. Both OH-PCB metabolites caused an increased phosphorylation of H2Ax. The proportion of cells with more than two foci correlated with the concentration of both metabolites. As in the comet assay, 3′-OHCB 28 showed a significant influence on the DNA integrity in Jurkat T cells at a concentration of 10 µM (see Fig. 4C). Both metabolites also led to an increase in the number of cells with more than five foci and thus increased DNA damage. With the densitometric evaluation of immunoblots, we used another method to quantify the formation of phosphorylated H2Ax (Fig. 4D). γH2Ax (15 kDa) was detected in total protein lysates of Jurkat T cells by using a phosphospecific antibody. The relative H2Ax phosphorylation was calculated upon the detection of ß-actin (42 kDa). Subsequently, the relative H2Ax phosphorylations were normalized on the negative control. Jurkat T cells showed a 5.5-fold higher phosphorylation of H2Ax as compared to negative controls when incubated for 48 hrs. with 10 µM 3-OHCB 28. Similar results were found for 3′-OHCB 28. Both metabolites showed a clear dose-response relationship for the phosphorylation of H2Ax. These findings therefore suggest that oxidation of PCB 28 produces DNA damage.

### Combination of OH-PCBs inhibits hTERT expression without reduction of metabolic activity

**Figure 5:**
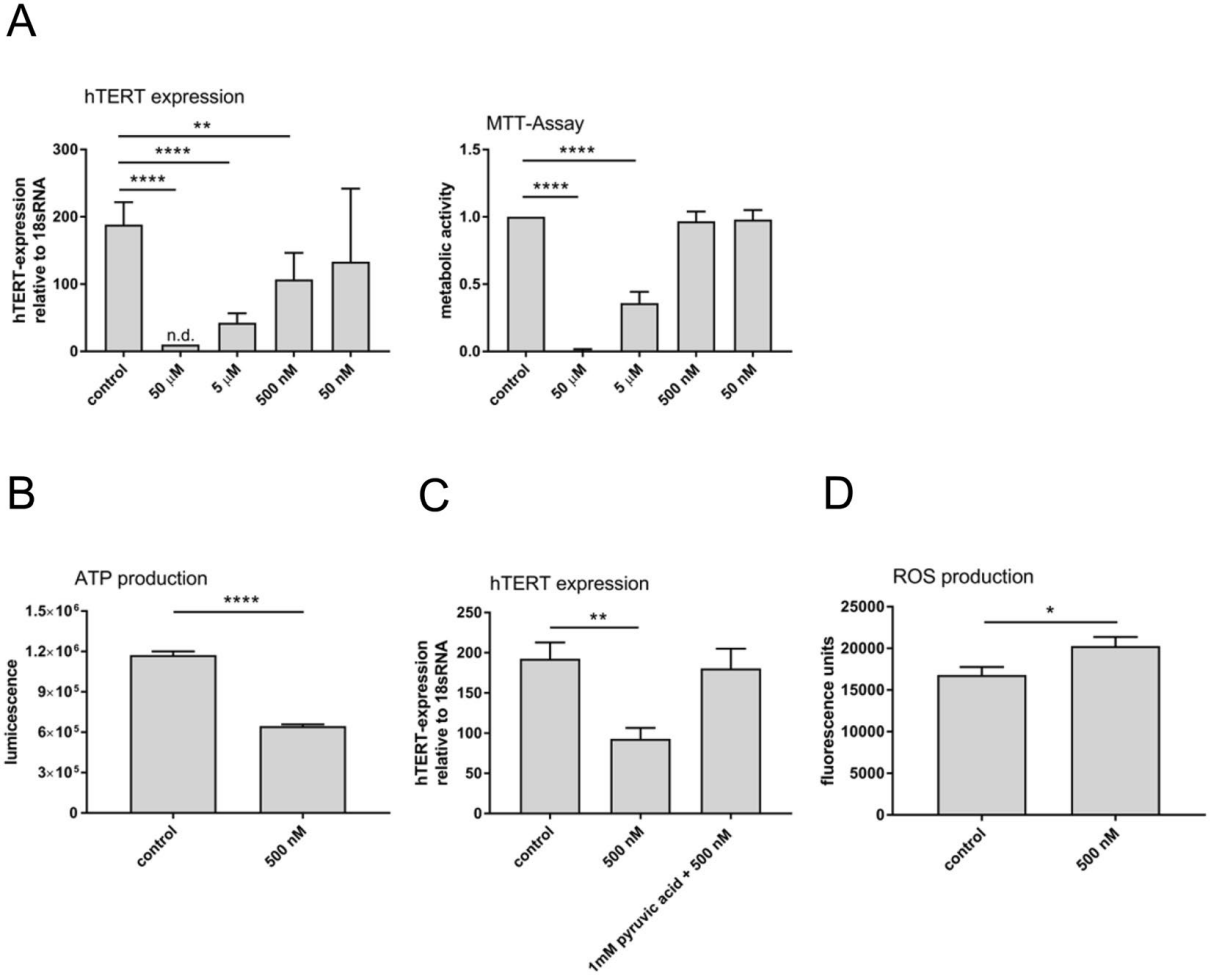
Combination of OH-PCBs inhibits hTERT expression without reduction of metabolic activity. (**A**) Left, Jurkat T cells were incubated with serial dilutions of a OH-PCB mix, containing equal amounts of 3-OHCB 28 and 3′-OHCB 28, as well as 3-OHCB 101 and 4-OHCB 101, for 48 hours. The following concentrations of the OH-PCB mix were used: 50 µM (Σ OH-PCB = 61.54 mg/L), 5 µM (Σ OH-PCB = 6.154 mg/L), 500 nM (Σ OH-PCB = 615.4 μg/L) and 50 nM (Σ OH-PCB = 61.54 µg/L). *hTERT* gene expression (left) and mitochondrial and cellular viability (right) were measured and evaluated as described in Fig. 3. Mean ± SD of three different experiments each with a total of three replicates are shown. Statistical analysis was performed by an unpaired, two-tailed Student’s t-test. Statistically significant differences are indicated (**P < 0.01; ****P < 0.0001). (**B**) Jurkat T cells were preincubated for 4 hrs. with 500 nM OH-metabolites mix. ATP production was measured as luminescent signal after adding ATP detection reagent from ToxGlo^TM^-assay. Mean ± SD of three replicates are shown. Statistical analysis was performed by an unpaired, two-tailed Student’s t-test. Statistically significant differences are indicated (****P < 0.0001). (**C**) *hTERT* gene expression in the presence and absence of pyruvic acid. Jurkat T cells were treated with 500 nM OH-metabolites mix for 48 hrs. Where indicated, 24 hrs. before treatment start, pyruvic acid was added to the culture medium. *hTERT* expression was evaluated by RT-PCR as described. Mean ± SD of three different experiments each with a total of three replicates are shown. Statistical analysis was performed by an unpaired, two-tailed Student’s t-test. Statistically significant differences are indicated (**P < 0.01). (**D**) Production of reactive oxygen species (ROS) in Jurkat T cells in the presence of OH-PCB mix. Jurkat T cells preincubated with 4,5-diaminofluorescein-2-diacetate (DAF-2DA) were treated with 500 nM OH-PCB mix (Σ OH-PCB = 615.4 µg/L) for 4 hours and ROS production was measured as fluorescence signal. Mean ± SD of three replicates are shown. Statistical analysis was performed by an unpaired, two-tailed Student’s t-test. Statistically significant differences are indicated (*P < 0.05).

The concentrations of the OH-PCBs used for the *in vitro* experiments so far were several orders of magnitude higher than the concentrations measured in the plasma of individuals of the HELPcB-cohort^27^. In addition, when using single OH-PCBs derived from PCB 28 and PCB 101, a spsecific effect on *hTERT* expression without reduction of metabolic activity was not evident (Fig. 3A,B). We therefore assessed if the combination of the metabolites (OH-PCB mix) is able to potentiate the effects on *hTERT* gene expression in serial 10x dilutions (Fig. 5A). Equal amounts of 3-OHCB 28 and 3′-OHCB 28, as well as 3-OHCB 101 and 4-OHCB 101 were mixed in equal concentrations starting at 50 µM per metabolite (Σ OH-PCB = 61.54 mg/L) and diluting to 5 µM (Σ OH-PCB = 6.154 mg/L), 500 nM (Σ OH-PCB = 615.4 μg/L) and 50 nM (Σ OH-PCB = 61.54 µg/L). At each concentration the inhibition of *hTERT* gene expression was tested by RT-PCR as described above. At concentrations of 50 µM and 5 µM, the OH-PCB mix almost completely inhibited the expression of *hTERT*. In addition, a significant 45% reduction of *hTERT* expression could be shown at 500 nM, whereas at 50 nM, inhibitory effects were evident but inconsistent (Fig. 5A). OH-PCB metabolites of LC PCBs can thus, in combination with each other, increase their respective effects on *hTERT* expression and reduce the overall bio effective PCB concentration at the same time. These results further indicate that the concentrations of OH-PCBs for *hTERT* inhibition *in vitro* are principally capable of matching the results obtained with blood plasma from the HELPcB-cohort *ex vivo*. As the effects of individual OH-PCBs on *hTERT* gene expression in Jurkat T-cells mostly reflected a lower number of living cells, we next assessed metabolic activity of Jurkat T cells incubated with the OH-PCB mix as described above (Fig. 5B). By using the OH-PCB mix and its dilutions, a significant inhibition of metabolic activity could be reached at 50 µM and 5 µM concentrations, respectively. Lower OH-PCB mix levels had no effect on metabolic activity in Jurkat T cells. Together with the experiments on inhibition of *hTERT* expression these results therefore show, that the combination of OH-PCBs inhibits *hTERT* expression at both toxic and subtoxic concentrations. In addition, a selective inhibition of *hTERT* at zero metabolic toxicity can be achieved by combining different OH-metabolites.

MTT assays are dependent on mitochondrial respiration and indirectly serve to assess the cellular energy capacity of a cell^28^. The overall strong effect of OH-PCBs in the MTT assay prompted us to investigate if LC PCBs could influence the function of mitochondria, e.g. the production of ATP, without reducing metabolic activity. Jurkat T cells were therefore incubated with a 500 nM (Σ OH-PCB = 615.4 μg/L) OH-PCB mix. With this concentration no effect on metabolic activity could be observed, but the inhibition of *hTERT* expression was still evident (Fig. 5A). After 1 hour of incubation, ATP production levels had dropped around 50% compared to control treated samples (Fig. 5B), implying that even at a low dose, OH-PCBs interfere with the mitochondrial respiratory chain and ATP synthesis. Since it is not clear, whether mitochondrial dysfunction causes the inhibition of *hTERT* expression, or whether *hTERT* inhibition is an independent event in LC PCB toxication, we next assessed if PCB mediated *hTERT* inhibition and mitochondrial damage are intertwined. Therefore Jurkat T cells were preincubated for 24 hours with 1 mM pyruvic acid, the key intermediate of several energy producing process reactions within mitochondria. After incubating cells for a further 48 hours with the 500 nM OH-PCB mix, *hTERT* gene expression was evaluated. Interestingly, pyruvic acid prevented the OH-PCB mix induced *hTERT* inhibition at 500 nM (Fig. 5C). Mitochondrial dysfunction and inhibition of *hTERT* expression induced by activation of LC PCBs seemed therefore to be part of the same energy stress-induced pathway.

For OH-metabolites derived from PCB 28 (3 chlorine atoms), oxidation to quinones or semiquinones is possible. A reactive semiquinone which, in the presence of O_2_, can form a reactive oxygen species (ROS), such as superoxide anions, could thus cause oxidative stress. In addition, OH-PCBs could interfere with the regulation of both intra-mitochondrial ROS production and disposal^10^. For that reason, we took a look at ROS production in the presence and absence of the 500 nM OH-PCB mix. A significant increase in ROS production in the presence of the mixture of OH-metabolites in comparison to the solvent control was observed (Fig. 5D). In summary these results show that similar to dioxin-like coplanar PCBs causing oxidative stress in the brain, PCB 28 and PCB 101 derived OH-PCBs are potent promoters of oxygen radical formation, which together with their derivatives can oxidize and alter important functional macromolecules within the cell.

## Discussion

The effect of PCBs on humans is very heterogeneous and depends on various factors such as the congeners involved, cell-types affected, the degree of exposure and genetic predisposition^6^. Low-chlorinated PCBs are more reactive than HC PCB and DL PCB and their metabolism can lead to intermediates (arene oxides) with a higher toxicity than their parent compounds. In addition, LC PCBs are suspected of causing mutations in genetic material^29,30^. Low-chlorinated, volatile PCBs from open systems (e. g. joint sealants) reach the air through permanent emission and can thus be incorporated into the human body by inhalation^31^. In addition, when replaced by recycling companies, improper handling and non-compliance with hygiene regulations can result in a considerable LC PCB impact on employees and their relatives^32^.

In this paper we report a dose-response linkage between the effects of LC PCBs and their concentrations in human plasma. Using *hTERT* gene expression bioassays we establish the highest doses at which no toxic effects were identified and the lowest doses at which toxic or adverse effects were observed. Usually, the three indicator congeners for LC PCBs (PCB 28, 52, 101) and three indicator congeners for the HC PCBs (PCB 138, 153, 180) are analyzed in human plasma samples. This selection is the established surrogate for estimating the internal PCB burden, but does not focus on toxicological aspects. Until now, plasma concentration-effect relationships for LC PCBs could never be analysed and only biological reference values were being specified. For instance, the German MAK collection on Occupational Health and Safety established a biological tolerance value at the work place (BAT value) at 15 μg/L for the combined plasma levels of the PCB congeners 28, 52, 101, 138, 153, and 180^33^. With the *hTERT* gene expression bioassay defining a NOAEL at 0.2–0.5 μg/L and a LOAEL between 0.5 and 2 μg/L LC PCB, we believe, that verifiably adverse health effect levels of LC PCBs begin to occur at a concentration of ≥ 0.5 µg/L blood plasma.

Our *in vitro* experiments using Jurkat T cells suggest, that the oxidation of LC PCBs to reactive OH-intermediates is a prerequisite for the effects on *hTERT* expression and cellular integrity. In fact, individual OH-metabolites derived from PCB 28 and PCB 101 (OH-metabolites of PCB52 are not defined in humans) already show a much stronger inhibition of *hTERT* gene expression at a lower concentration than their parental compounds (Fig. 3A). This can be further enhanced by the combination of the metabolites 3-OHCB 28 and 3′-OHCB 28, as well as 3-OHCB 101 and 4-OHCB 101 (Fig. 5A).

As we have observed significant positive correlations between PCB 28 and its metabolites in the plasma of the HELPcB cohort^12^, the *hTERT* gene expression bioassay described in this study seems to indicate metabolic activation of LC PCBs rather than the mere effect of LC PCBs on their own. Therefore the dependence of the production of reactive OH-metabolites on the level of LC PCB exposure should be taken into account in future dose-response assessments. For instance the concentration of LC PCB at which saturation of the involved cytochrome P450-enzyme begins should be clarified and attention should be paid to the fact, that some OH-PCBs show higher half-lives than their parental PCB^12^. Moreover, CYP450 monooxygenases are in fact characterized by more frequent polymorphisms^34^, which lead to stronger or weaker active or inactive variants of the enzymes. Some PCB exposed individuals therefore might be more rapid converters of LC PCBs than others.

Several bioassays for the detection of PCBs have been described in the literature. Samples suspected to contain polychlorinated dibenzo- para dioxins (PCDD), dibenzofurans (PCDF) or DL PCBs can be screened with EROD activity based and CALUX test systems, respectively. In both bioassays, DL PCBs bind to the cellular aryl hydrocarbon (Ah) receptor^35^. This leads to an induction of the CYP1A1 gene in the EROD assay. The 7-etoxyresorufin-o-deethylase (EROD) formed in this process, which is associated with the CYP1A1 enzyme, converts the added substrate 7-ethoxyresorufin into a measurable fluorescent resorufin. The CALUX DR assay is a reporter gene assay in which a DNA construct consisting of a dioxin response element (DRE) promoter and a downstream luciferase reporter gene have been stably integrated into the genome of the rat liver cell line H4IIE^35^. The observed luciferase expression is directly proportional to the binding of dioxin to the Ah receptor. Whereas EROD and CALUX assays are able to measure contamination within a diverse array of applications (e.g. sediment, soil, water, tissue, food, feedstuff), the *hTERT* gene expression bioassays described in this study, report promoter activity due to the metabolic activation of a pollutant (LC PCB) to reactive intermediates. Most importantly, these assays are able to generate signals from an amount of toxic OH-PCBs which is too low to induce any measurable effect on metabolic activity (see Fig. 5A). Whereas many *in vitro* studies on LC PCBs and their metabolites suffer consistently from the very high concentrations used, which are in range of cytotoxic concentrations, this assay is able to detect genotoxic substances at a concentration range that is actually also reached in humans. With the assays described in this study we add a new tool for the differentiated analysis of PCB congeners and their metabolites. Our results thus contribute to the risk assessment of PCB-related diseases. In addition to the general assessment of the exposure to PCBs and their toxic potency, the knowledge of interindividual differences in metabolizing capacity with respect to PCBs allows to include an individual component of the person concerned in the assessment.

It is well established that mammalian telomerase expression is controlled at multiple levels with transcription factor binding and regulation of chromatin playing key roles^36^. It would therefore not come as a surprise that other plasma components of the HELPcB cohort intervene in these regulation mechanisms and therefore increase the inhibitory effect of LC PCBs. Both HC PCB and DL PCB have been shown to interfere with telomerase activity and the telomere complex^19^, which could enhance the effects of LC PCB derived OH-metabolites on *hTERT* inhibition. In addition, endogenous substances, whose production or release is induced by PCBs, can have an effect on the expression of *hTERT*. Thus, prostaglandins reduce the activity of telomerase and accelerate cellular ageing^37^. LC PCBs have been shown to increase the production of prostaglandins *in vitro* and *in vivo*^38,39^ and thus could indirectly inhibit telomerase *hTERT* gene expression. In fact, prostaglandin levels in the plasma of the HELPcB cohort are increased by a factor of 4 compared to control plasma samples (data not shown).

PCBs have been shown to induce mitochondrial dysfunction and oxidative stress *in vitro*^40^ and upon application *in vivo*, reduce the surfaces and volume density of mitochondria while maintaining their numbers at a constant rate. As a consequence, the energy supply of mitochondria were reduced, as could be demonstrated by incubating Jurkat T cells with OH-PCBs at a concentration of 500 nM (Fig. 5B). Mitochondrial dysfunction can also be induced by glucose restriction^41^, which modulates *hTERT* gene expression by DNA methylation changes and/or histone remodeling^42^. Thus, inhibition of *hTERT* expression by OH-metabolites of LC PCBs could reflect a reduced activity of the mitochondrial respiratory chain. This assumption is supported by the protective effect of pyruvic acid, which was added to the culture medium. Pyruvic acid as a key regulator of metabolism and aerobic energy production, prevented *hTERT* inhibition by the OH-PCB mix most likely by restoration of oxidative phosphorylation and aerobic glycolysis. This could provide evidence against a mechanism involving covalent binding of OH-PCB derived quinones with cytochrome c, an important electron acceptor within the mitochondrial respiratory chain^43^. In contrast to that, oxidation of the PCB 28 metabolites 3-OHCB 28 and 3′-OHCB 28 to quinones or semiquinones would be a prerequisite for DNA adduct formation as observed for PCB 28 after incubation of isolated DNA with rat microsomes^44^.

## Material and Methods

### Study groups

Blood samples from the PCB-exposed HELPcB-cohort were collected in longitudinal studies as described previously^27,45^. Blood samples from rresidents exposed to PCBs via indoor air were collected between September 2010 and March 2014 as part of human biomonitoring studies. Exposure to lower chlorinated PCBs within the resident-cohort was verified by air monitoring in respective buildings, which partially exceeded a preventive threshold value of 300 ng/m^3^ air. Studies were carried out according to the Helsinki Declaration recommendations, participation was voluntary and all participants provided their written informed consent regarding the donation of blood and urine samples for scientific purposes. An approval by the ethical commission of the RWTH Aachen University is available (Nr. EK 176/11). Details of the internal PCB burden for each congener and the sum of non-dioxin-like PCB for the HELPcB-cohort and the resident-cohort are shown in Supplementary Tables 1–3.

### Cell culture and incubation with human blood plasma

Jurkat T cells (DSMZ, Germany) were cultured in RPMI 1640-GlutaMAX™-I supplemented with 10% (vol/vol) fetal calf serum (FCS), penicillin (50 U/mL) and streptomycin (50 μg/ml). The chromosomal BAC reporter fibroblast cell line tert^+^ B6B5.1 (gift of Jiyue Zhu, Department of Cellular and Molecular Physiology, Pennsylvania State University) was cultured in MEMα GlutaMAX™ supplemented with 10% (vol/vol) fetal calf serum (FCS), penicillin (50 U/mL) and streptomycin (50 μg/mL). Cells were passaged using Trypsin-EDTA (0.25%) once a week. For incubation with blood plasma from PCB exposed individuals, plasma samples were taken from frozen stocks, thawed and filtrated. Cells were cultured in a standard medium for 48 hrs. in the presence of 20% (vol/vol) human plasma. Plasma samples collected in 2011 and 2015 from the same individual were tested in parallel.

### Peripheral blood mononuclear cell (PBMC) separation and lymphoproliferation assay

Culture and expansion of primary cytotoxic T cells was performed as described previously^20^. Forty milliliters of peripheral blood were collected after informed consent from healthy donors. PBMCs were enriched by density gradient centrifugation at 500 *g* for 45 minutes at room temperature using Percoll Hypaque. After two rounds of washing with phosphate-buffered saline (PBS), cells were put in a culture with a concentration of 1 × 10^7^ cells per 2 mL of medium. The medium used throughout the experiments was RPMI 1640-GlutaMAX™-I supplemented with 10% (vol/vol) fetal calf serum (FCS), penicillin (50 U/mL) and streptomycin (50 μg/mL). T cells were stimulated in the presence of interleukin 2 and 7 (IL-2; IL-7; 40 IU/mL; PeproTech Inc, Rocky Hill, CT, USA) and tetanus toxoid (3 μg/mL, Statens Serum Institute, Copenhagen, Sweden). After 5 days of incubation cells were harvested, reseeded and used for experimentation.

### *hTERT* gene expression

Luciferase assays of the chromosomal BAC reporter cell line tert^+^ B6B5.1 were measured in triplicate using the Dual Luciferase Reporter assay system (Promega, Mannheim, Germany). qRT-PCR was performed as described before^20^. Jurkat T cells and proliferating PBMCs were lysed in TRIzol reagent, and RNA was purified according to the manufacturer’s instructions (Invitrogen, Carlsbad, USA). cDNA was synthesized using random hexamers primer and Superscript III reverse transcriptase according to the manufacturer’s instructions (Invitrogen, Carlsbad, CA, USA). Equal amounts of total RNA from various samples was used for RT-PCR reactions. Primers for telomerase (*hTERT*) and 18S rRNA were obtained from Invitrogen (Assay-on-Demand Gene expression reagents; Invitrogen; Carlsbad, CA, USA). All experiments were repeated at least once in triplicate. For studying the effects of PCB contaminated plasma on *hTERT* expression (Fig. 1B) by RT-PCR, serial dilutions of standard DNA were run in parallel. The relative telomerase to 18S rRNA ratio was calculated by dividing the copy number of the telomerase template by the copy number of the 18S rRNA template. For all other RT-PCR experiments, expression of *hTERT* was calculated according to the 2^−ΔΔCT^ method. cDNA was amplified using a sequence detector (7500 Fast Real-Time PCR System; Invitrogen, Carlsbad, CA, USA) and TaqMan target mixes (Assay-on-Demand Gene expression reagents; Invitrogen; Carlsbad, CA, USA).

### Cytotoxicity assays

Cytotoxicity assays were performed as described previously^20^. Jurkat T cells were cultured in the presence of increasing concentrations of parent PCBs (LGC, UK; purity PCB 28: 99.4%, PCB 101: 96,1%) or single OH-PCBs (custom synthesized at the Max Planck Institute for Biophysical Chemistry, Göttingen, Germany) or OH-PCB mixtures dissolved in either ethanol or isooctane for 48 hrs. The final concentration of the solvent in the medium amounts to maximal 2% (vol/vol). For the determination of metabolic activity, cells were plated into 96-well flat-bottomed microtiter plates (Becton Dickinson, Heidelberg, Germany) at a density of 1 × 10^5^ cells in 100 μL of media. Cells were grown for 24 hours before PCBs were added. 48 hours later the ability of remaining viable cells to transform 3-(4,5-dimethylthiazol-2-yl)-2,5-diphenyltetrazolium bromide (MTT) into formazan was assessed (MTT Cell Proliferation Assay, ATCC, Manassas, USA). The absorbance of the samples was measured on a Microplate Reader (FLUOstar, BMG Labtech, Ortenberg, Germany) at 450 nm.

### ATP-production and ROS-assays

ATP amount was measured using a commercial bioluminescent assay (ToxGlo^TM^, Promega, Mannheim, Germany) in a standard luminometer (Infinite® 200 PRO, Tecan, Zurich, Switzerland). For ROS production Jurkat T cells were incubated with 10 μM 4,5-diaminofluorescein-2-diacetate (DAF-2DA) in a humidified 5% CO_2_ atmosphere at 37 °C for 60 min and then treated with 500 nM OH-PCB mix for 4 hours. Fluorescence was assessed at an excitation/emission wavelength of 480/530 nm with a standard luminometer (Infinite® 200 PRO, Tecan, Zurich, Switzerland) every five minutes for one hour.

### Comet-assay

For neutral comet assays, Jurkat T cells were treated with parent PCBs or single OH-PCBs. After treatments 50 μL of a suspension of 10^5^ cells/mL were washed in PBS and resuspended in 500 μl of preheated agarose (37 °C). The agarose/cell suspensions were applied to microscopic slides and left for 30 minutes at 4 °C to allow gel polymerisation. After cell lysis using a commercial buffer (Trevigen, Gaithersburg, USA), single cell gelelectrophoresis was performed at 1 V/cm for 1 hour. After precipitation of the DNA with ammonium acetate, samples were fixed in 70% ethanol for 30 minutes and dried. To visualize dsDNA SYBR™ Green I Nucleic Acid Gel Stain (Invitrogen, Carlsbad, USA) was used. Pictures of at least 50 cells/section were taken using a fluorescence microscope (DMRX, Leica: 450–490 nm BP- filter, 40x magnification). The extent of the genotoxic effect of PCB metabolites was calculated using the ImageJ-based OpenComet software.

### Detection of DNA damage response

The detection of a DNA damage response was performed by analyzing phosphorylation of the histone H2Ax by indirect fluorescence microscopy or immune-blotting. For microscopy, Jurkat T cells were grown on polylysine-coated glass cover slips. After incubation with PCBs, cells were fixed with methanol-acetic acid (3:1) and immunostaining was performed according to standard procedures. The primary anti-phospho-H2Ax antibody (Merck Millipore, Burlington, MA, USA, Cat. # 05-636, clone JBW301) was diluted 1:200 and applied over night at 4 °C, followed by secondary Alexa Fluor-488-conjugated antibody (Invitrogen, Carlsbad, USA) 1:200 and applied for 60 min. The cover slips were mounted with ImmuMount (Thermo Scientific, Pittsburgh, PA, USA), containing DAPI. Fluorescence and DIC images were generated with a Zeiss LSM 710 confocal microscope (Zeiss, Jena, Germany). Adherent cells were examined with a Zeiss LD C-apochromat 40×/1.1 water objective. Intensity profiles were generated with the ZEN 2009 software (Zeiss, Jena, Germany).

For immune-blotting, cells were lysed, proteins separated by SDS-PAGE and transferred to a polyvinylidene fluoride membrane (PALL, Dreieich, Germany) according to standard protocols. The membrane was blocked with Roti R-Block overnight and incubated with the primary anti-phospho-H2Ax antibody (Merck Millipore, Burlington, MA, USA, Cat. # 05-636, clone JBW301) diluted 1:2000. The membrane was washed three times with a TBST buffer (200 mM Tris(hydroxymethyl)aminomethane, 1.5 M NaCl, 1% (v/v) Tween-20, pH 7,5) for 15 min and treated with a suitable secondary Anti-mouse IgG antibody conjugated to horseradish peroxidase (Vector Laboratories, Burlingame, CA, USA, Cat. # PI-2000) diluted 1:5000 for 1 h. β-Actin served as control and was detected with an Anti-β-Actin antibody (Sigma-Aldrich, St. Louis, MO, USA, Cat. # A1978) diluted 1:4000. Bound antibodies were detected by chemiluminescence (ECL, Merck Millipore, Burlington, MA, USA).

### Statistical analysis

Statistical analysis was done by a Wilcoxon matched-pairs signed rank test for telomerase gene expression assays performed with PCB plasma samples from 2011 and 2015 (Fig. 1A–C). Analysis of concentration-dependent differences in telomerase gene expression was done by a Mann-Whitney test (Figs 1D and 2A). For all other data sets two-tailed, unpaired Student’s t-tests were performed. Where indicated, differences were investigated using Kruskal-Wallis followed by a Dunn’s post-hoc test for multiple comparisons (Fig. 4A). All statistical tests performed are designated in the respective figure legends.

## Electronic supplementary material

Supplementary Dataset 1

## Data Availability

The data generated and/or analysed during the current study are available from the corresponding author on request.
