# Supplementary material for: Telomerase gene expression bioassays indicate metabolic activation of genotoxic lower chlorinated polychlorinated biphenyls
Source: Sci Rep. 2018 Nov 15;8:16903. doi: 10.1038/s41598-018-35043-w (PMC6237825; doi:10.1038/s41598-018-35043-w)

**Supplementary information to:**

**Telomerase gene expression bioassays indicate metabolic activation of genotoxic lower chlorinated polychlorinated biphenyls**

Theresa Vasko^1^, Jenny Hoffmann^1^, Sonja Gostek^1^, Thomas Schettgen^1^, Natalia Quinete^1,2^, Christian Preisinger^3^, Thomas Kraus^1^ and Patrick Ziegler^1^

1. Institute for Occupational, Social and Environmental Medicine, RWTH Aachen University, Aachen, Germany

2. Southeast Environmental Research Center, Florida International University Florida, USA.

3. Proteomics Facility, IZKF, RWTH Aachen University, Aachen, Germany

Supplemental Tables

**Supplementary Table 1: Participants**

|  | **N** | **Mean** | **Median** | **SD** | **Minimum** | **Maximum** | **25^th^Perc.** | **50^th^Perc.** | **75^th^Perc.** | **95^th^Perc.** |
| --- | --- | --- | --- | --- | --- | --- | --- | --- | --- | --- |
| **Female** | 15 | 46.4 | 47.8 | 10.3 | 27.5 | 60.6 | 42.8 | 47.8 | 53.1 | 58.5 |
| **Male** | 77 | 46.8 | 46.3 | 12.5 | 23.1 | 84.3 | 38.0 | 46.3 | 57.5 | 62.5 |
| **Total** | 92 | 46.7 | 47.6 | 12.2 | 23.1 | 84.3 | 39.3 | 47.6 | 57.0 | 62.3 |

Table 1a: Age distribution by gender within HELPcB-cohort 2011

|  | **N** | **Mean** | **Median** | **SD** | **Minimum** | **Maximum** | **25^th^Perc.** | **50^th^Perc.** | **75^th^Perc.** | **95^th^Perc.** |
| --- | --- | --- | --- | --- | --- | --- | --- | --- | --- | --- |
| **Female** | 15 | 50.3 | 51.6 | 10.3 | 31.5 | 64.6 | 46.8 | 51.6 | 57.1 | 62.5 |
| **Male** | 77 | 50.7 | 50.2 | 12.4 | 27.1 | 88.3 | 41.9 | 50.2 | 61.1 | 66.4 |
| **Total** | 92 | 50.6 | 51.4 | 12.1 | 27.1 | 88.3 | 43.2 | 51.4 | 60.7 | 66.3 |

Table 1b: Age distribution by gender within HELPcB-cohort 2015

|  | **N** | **Mean** | **Median** | **SD** | **Minimum** | **Maximum** | **25^th^Perc.** | **50^th^Perc.** | **75^th^Perc.** | **95^th^Perc.** |
| --- | --- | --- | --- | --- | --- | --- | --- | --- | --- | --- |
| **Female** | 56 | 43.7 | 45.3 | 13.8 | 18.7 | 67.9 | 30.2 | 45.3 | 56.2 | 63.3 |
| **Male** | 46 | 42.4 | 45.0 | 14.0 | 24.6 | 70.3 | 27.5 | 45.0 | 52.9 | 64.1 |
| **Total** | 102 | 43.1 | 45.0 | 13.9 | 18.7 | 70.3 | 29.3 | 45.0 | 55.4 | 64.0 |

Table 1c: Age distribution by gender within indoor-cohort

**Supplementary Table 2: Distribution of non-dioxin-like PCB congeners within cohorts**

|  | **PCB 28** | **PCB 52** | **PCB 101** | **PCB ∑ lc** | **PCB 138** | **PCB 153** | **PCB 180** | **PCB ∑ ndl** |
| --- | --- | --- | --- | --- | --- | --- | --- | --- |
| **N valid** | 92 | 92 | 92 | 92 | 92 | 92 | 92 | 92 |
| **N missing** | 0 | 0 | 0 | 0 | 0 | 0 | 0 | 0 |
| **Mean** | 0.629 | 0.104 | 0.222 | 0.954 | 2.047 | 2.147 | 1.359 | 6.507 |
| **Median** | 0.054 | 0.005 | 0.019 | 0.079 | 0.699 | 0.945 | 0.782 | 2.634 |
| **SD** | 2.582 | 0.680 | 1.365 | 3.397 | 3.467 | 3.102 | 1.843 | 10.253 |
| **Minimum** | 0.005 | 0.005 | 0.005 | 0.015 | 0.101 | 0.129 | 0.081 | 0.358 |
| **maximum** | 23.957 | 6.552 | 13.127 | 25.220 | 22.124 | 16.225 | 10.238 | 65.533 |
| **25^th^Perc.** | 0.024 | 0.005 | 0.005 | 0.035 | 0.401 | 0.538 | 0.369 | 1.547 |
| **50^th^Perc.** | 0.054 | 0.005 | 0.019 | 0.079 | 0.699 | 0.945 | 0.782 | 2.634 |
| **75^th^Perc.** | 0.305 | 0.025 | 0.049 | 0.426 | 2.066 | 2.165 | 1.259 | 6.046 |
| **95^th^Perc.** | 1.936 | 0.198 | 0.384 | 2.859 | 8.596 | 9.054 | 5.409 | 26.120 |

Table 2a: Distribution of non-dioxin like (ndl) PCB congeners with sum of lower chlorinated (lc) PCB 28, PCB 52 and PCB 101 and the sum of all non-dioxin like PCBs within HELPcB-cohort 2011

|  | **PCB 28** | **PCB 52** | **PCB 101** | **PCB ∑ lc** | **PCB 138** | **PCB 153** | **PCB 180** | **PCB ∑ ndl** |
| --- | --- | --- | --- | --- | --- | --- | --- | --- |
| **N valid** | 92 | 92 | 92 | 92 | 92 | 92 | 92 | 92 |
| **N missing** | 0 | 0 | 0 | 0 | 0 | 0 | 0 | 0 |
| **Mean** | 0.221 | 0.007 | 0.017 | 0.245 | 1.821 | 2.098 | 1.445 | 5.613 |
| **Median** | 0.014 | 0.005 | 0.005 | 0.032 | 0.643 | 0.818 | 0.766 | 2.240 |
| **SD** | 0.895 | 0.007 | 0.030 | 0.919 | 2.872 | 3.025 | 1.960 | 8.033 |
| **Minimum** | 0.005 | 0.005 | 0.005 | 0.015 | 0.076 | 0.109 | 0.067 | 0.266 |
| **Maximum** | 7.716 | 0.051 | 0.233 | 7.859 | 15.723 | 15.911 | 9.500 | 42.306 |
| **25^th^Perc.** | 0.005 | 0.005 | 0.005 | 0.015 | 0.384 | 0.550 | 0.410 | 1.469 |
| **50^th^Perc.** | 0.014 | 0.005 | 0.005 | 0.032 | 0.643 | 0.818 | 0.766 | 2.240 |
| **75^th^Perc.** | 0.098 | 0.005 | 0.015 | 0.131 | 1.944 | 2.366 | 1.458 | 6.295 |
| **95^th^Perc.** | 0.575 | 0.018 | 0.074 | 0.635 | 6.721 | 7.246 | 6.070 | 20.486 |

Table 2b: Distribution of non-dioxin like (ndl) PCB congeners with sum of lower chlorinated (lc) PCB 28, PCB 52 and PCB 101 and the sum of all non-dioxin like PCBs within HELPcB-cohort 2015

|  | **PCB 28** | **PCB 52** | **PCB 101** | **PCB ∑ lc** | **PCB 138** | **PCB 153** | **PCB 180** | **PCB ∑ ndl** |
| --- | --- | --- | --- | --- | --- | --- | --- | --- |
| **N valid** | 102 | 102 | 102 | 102 | 102 | 102 | 102 | 102 |
| **N missing** | 0 | 0 | 0 | 0 | 0 | 0 | 0 | 0 |
| **Mean** | 0.069 | 0.020 | 0.014 | 0.103 | 0.342 | 0.553 | 0.454 | 1.451 |
| **Median** | 0.033 | 0.013 | 0.005 | 0.052 | 0.254 | 0.413 | 0.288 | 1.115 |
| **SD** | 0.085 | 0.024 | 0.015 | 0.117 | 0.290 | 0.495 | 0.461 | 1.297 |
| **Minimum** | 0.005 | 0.005 | 0.005 | 0.015 | 0.040 | 0.054 | 0.028 | 0.137 |
| **Maximum** | 0.489 | 0.176 | 0.078 | 0.710 | 1.504 | 2.637 | 2.669 | 6.940 |
| **25^th^Perc.** | 0.012 | 0.005 | 0.005 | 0.025 | 0.123 | 0.171 | 0.105 | 0.424 |
| **50^th^Perc.** | 0.033 | 0.013 | 0.005 | 0.052 | 0.254 | 0.413 | 0.288 | 1.115 |
| **75^th^Perc.** | 0.107 | 0.024 | 0.017 | 0.157 | 0.491 | 0.763 | 0.644 | 2.035 |
| **95^th^Perc.** | 0.175 | 0.052 | 0.047 | 0.276 | 0.858 | 1.368 | 1.277 | 3.677 |

Table 2c: Distribution of non-dioxin like (ndl) PCB congeners with sum of lower chlorinated (lc) PCB 28, PCB 52 and PCB 101 and the sum of all non-dioxin like PCBs within indoor-cohort

**Supplementary Table 3: Distribution of dioxin-like PCB congeners within cohorts**

|  | **PCB 77** | **PCB 81** | **PCB 105** | **PCB 114** | **PCB 118** | **PCB 123** | **PCB 126** | **PCB 156** | **PCB 157** | **PCB 167** | **PCB 169** | **PCB 189** |
| --- | --- | --- | --- | --- | --- | --- | --- | --- | --- | --- | --- | --- |
| **N valid** | 92 | 92 | 92 | 92 | 92 | 92 | 92 | 92 | 92 | 92 | 92 | 92 |
| **N missing** | 0 | 0 | 0 | 0 | 0 | 0 | 0 | 0 | 0 | 0 | 0 | 0 |
| **Mean** | 0.005 | 0.005 | 0.297 | 0.038 | 0.932 | 0.016 | 0.005 | 0.266 | 0.049 | 0.099 | 0.005 | 0.029 |
| **Median** | 0.005 | 0.005 | 0.045 | 0.012 | 0.200 | 0.005 | 0.005 | 0.103 | 0.019 | 0.030 | 0.005 | 0.017 |
| **SD** | 0.002 | 0.002 | 0.801 | 0.066 | 2.197 | 0.042 | 0.001 | 0.455 | 0.093 | 0.181 | 0.000 | 0.042 |
| **Minimum** | 0.005 | 0.005 | 0.005 | 0.005 | 0.019 | 0.005 | 0.005 | 0.011 | 0.005 | 0.005 | 0.005 | 0.005 |
| **Maximum** | 0.028 | 0.028 | 5.864 | 0.406 | 16.893 | 0.337 | 0.012 | 2.576 | 0.697 | 1.308 | 0.005 | 0.242 |
| **25^th^Perc.** | 0.005 | 0.005 | 0.019 | 0.005 | 0.084 | 0.005 | 0.005 | 0.058 | 0.011 | 0.019 | 0.005 | 0.005 |
| **50^th^Perc.** | 0.005 | 0.005 | 0.045 | 0.012 | 0.200 | 0.005 | 0.005 | 0.103 | 0.019 | 0.030 | 0.005 | 0.017 |
| **75^th^Perc.** | 0.005 | 0.005 | 0.165 | 0.031 | 0.691 | 0.005 | 0.005 | 0.225 | 0.040 | 0.093 | 0.005 | 0.026 |
| **95^th^Perc.** | 0.005 | 0.005 | 0.988 | 0.160 | 3.640 | 0.046 | 0.005 | 1.062 | 0.182 | 0.379 | 0.005 | 0.099 |

Table 3a: Distribution of dioxin like PCB congeners within HELPcB-cohort 2011

|  | **PCB 77** | **PCB 81** | **PCB 105** | **PCB 114** | **PCB 118** | **PCB 123** | **PCB 126** | **PCB 156** | **PCB 157** | **PCB 167** | **PCB 169** | **PCB 189** |
| --- | --- | --- | --- | --- | --- | --- | --- | --- | --- | --- | --- | --- |
| **N valid** | 92 | 92 | 92 | 92 | 92 | 92 | 92 | 92 | 92 | 92 | 92 | 92 |
| **N missing** | 0 | 0 | 0 | 0 | 0 | 0 | 0 | 0 | 0 | 0 | 0 | 0 |
| **Mean** | 0.005 | 0.005 | 0.124 | 0.030 | 0.484 | 0.008 | 0.005 | 0.281 | 0.047 | 0.090 | 0.005 | 0.032 |
| **Median** | 0.005 | 0.005 | 0.030 | 0.005 | 0.140 | 0.005 | 0.005 | 0.074 | 0.014 | 0.030 | 0.005 | 0.013 |
| **SD** | 0.000 | 0.000 | 0.302 | 0.060 | 1.006 | 0.011 | 0.000 | 0.544 | 0.091 | 0.152 | 0.000 | 0.055 |
| **Minimum** | 0.005 | 0.005 | 0.005 | 0.005 | 0.014 | 0.005 | 0.005 | 0.005 | 0.005 | 0.005 | 0.005 | 0.005 |
| **Maximum** | 0.005 | 0.005 | 1.912 | 0.341 | 6.321 | 0.086 | 0.005 | 3.318 | 0.573 | 0.791 | 0.005 | 0.314 |
| **25^th^Perc.** | 0.005 | 0.005 | 0.005 | 0.005 | 0.056 | 0.005 | 0.005 | 0.055 | 0.005 | 0.015 | 0.005 | 0.005 |
| **50^th^Perc.** | 0.005 | 0.005 | 0.030 | 0.005 | 0.140 | 0.005 | 0.005 | 0.074 | 0.014 | 0.030 | 0.005 | 0.013 |
| **75^th^Perc.** | 0.005 | 0.005 | 0.092 | 0.022 | 0.435 | 0.005 | 0.005 | 0.198 | 0.034 | 0.085 | 0.005 | 0.026 |
| **95^th^Perc.** | 0.005 | 0.005 | 0.398 | 0.146 | 1.853 | 0.021 | 0.005 | 1.357 | 0.219 | 0.373 | 0.005 | 0.140 |

Table 3b: Distribution of dioxin like PCB congeners within HELPcB-cohort 2015

|  | **PCB 77** | **PCB 81** | **PCB 105** | **PCB 114** | **PCB 118** | **PCB 123** | **PCB 126** | **PCB 156** | **PCB 157** | **PCB 167** | **PCB 169** | **PCB 189** |
| --- | --- | --- | --- | --- | --- | --- | --- | --- | --- | --- | --- | --- |
| **N valid** | 102 | 102 | 102 | 102 | 102 | 102 | 102 | 102 | 102 | 102 | 102 | 102 |
| **N missing** | 0 | 0 | 0 | 0 | 0 | 0 | 0 | 0 | 0 | 0 | 0 | 0 |
| **Mean** | 0.005 | 0.005 | 0.013 | 0.006 | 0.072 | 0.005 | 0.005 | 0.054 | 0.010 | 0.016 | 0.005 | 0.010 |
| **Median** | 0.005 | 0.005 | 0.005 | 0.005 | 0.053 | 0.005 | 0.005 | 0.045 | 0.005 | 0.012 | 0.005 | 0.005 |
| **SD** | 0.000 | 0.000 | 0.012 | 0.004 | 0.060 | 0.000 | 0.000 | 0.054 | 0.008 | 0.014 | 0.000 | 0.009 |
| **Minimum** | 0.005 | 0.005 | 0.005 | 0.005 | 0.005 | 0.005 | 0.005 | 0.005 | 0.005 | 0.005 | 0.005 | 0.005 |
| **Maximum** | 0.005 | 0.005 | 0.063 | 0.028 | 0.307 | 0.005 | 0.005 | 0.311 | 0.047 | 0.071 | 0.005 | 0.055 |
| **25^th^Perc.** | 0.005 | 0.005 | 0.005 | 0.005 | 0.026 | 0.005 | 0.005 | 0.014 | 0.005 | 0.005 | 0.005 | 0.005 |
| **50^th^Perc.** | 0.005 | 0.005 | 0.005 | 0.005 | 0.053 | 0.005 | 0.005 | 0.045 | 0.005 | 0.012 | 0.005 | 0.005 |
| **75^th^Perc.** | 0.005 | 0.005 | 0.018 | 0.005 | 0.107 | 0.005 | 0.005 | 0.075 | 0.014 | 0.023 | 0.005 | 0.013 |
| **95^th^Perc.** | 0.005 | 0.005 | 0.037 | 0.013 | 0.203 | 0.005 | 0.005 | 0.140 | 0.026 | 0.043 | 0.005 | 0.025 |

Table 3c: Distribution of dioxin like PCB congeners within indoor-cohort

**Supplementary Fig. 1:** Structural formulas of OH-PCBs used in this study.

3-OH-CB28

3-OH-CB101

4-OH-CB101

3´-OH-CB28

PCB28

Cl

Cl

Cl

Cl

Cl

Cl

HO

Cl

Cl

Cl

OH

Cl

Cl

Cl

OH

4´-OH-CB31

Cl

Cl

Cl

HO

4-OH-CB25

Cl

Cl

Cl

OH

Cl

Cl

PCB101

Cl

Cl

Cl

Cl

Cl

Cl

Cl

Cl

OH

Cl

Cl

**Supplementary Fig. 2:** Full-length Western Blot H2Ax. Exposure time: 50 seconds. Last sample was not part of the experiment.


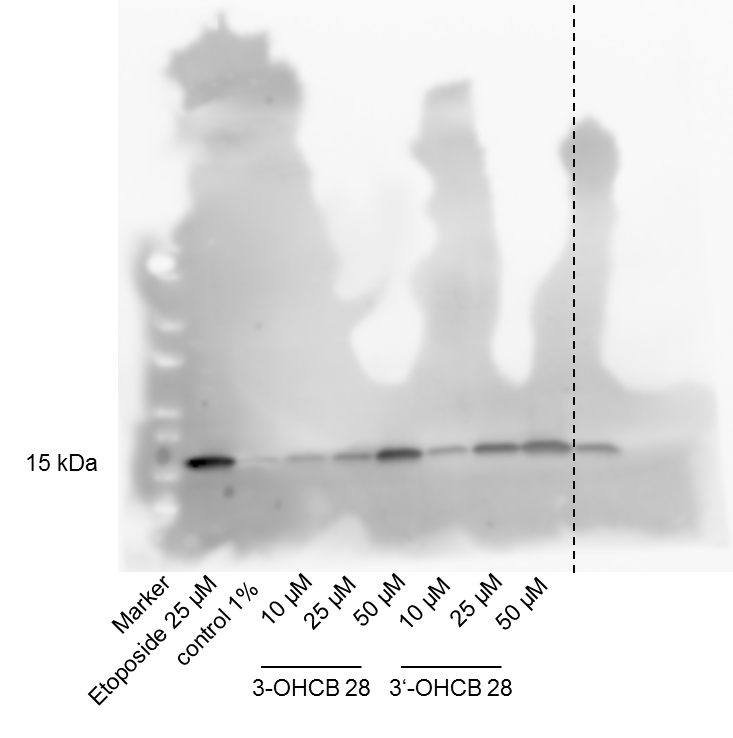


**Supplementary Fig. 3:** Full-length Western Blot β-Actin (stripped after γH2Ax detection). Exposure time: 110 seconds. Last sample was not part of the experiment.


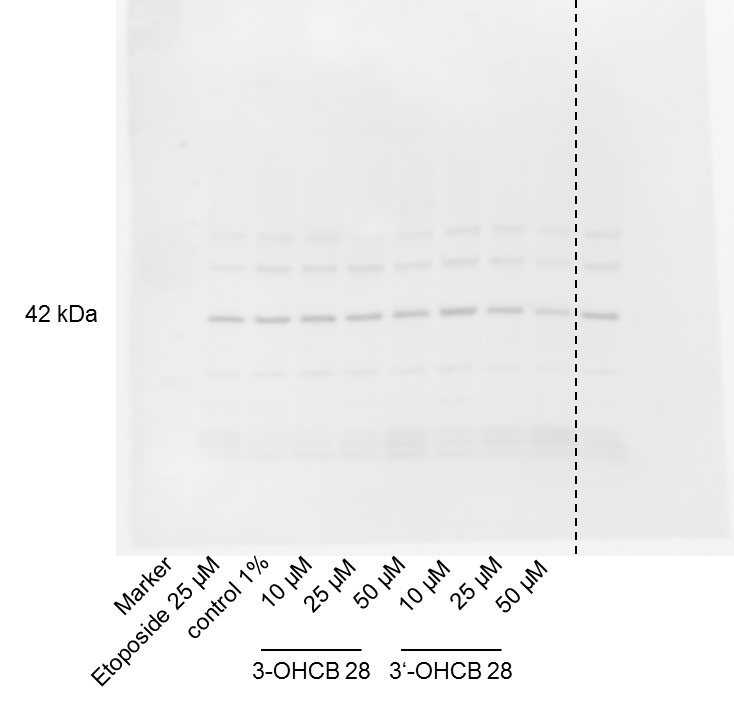

Supplement: Supplementary file 1 — Supplementary Dataset 1 [file 41598_2018_35043_MOESM1_ESM.docx]
